# Chronic Pain Diagnosis Using Machine Learning, Questionnaires, and QST: A Sensitivity Experiment

**DOI:** 10.3390/diagnostics10110958

**Published:** 2020-11-17

**Authors:** Alex Novaes Santana, Charles Novaes de Santana, Pedro Montoya

**Affiliations:** Research Institute of Health Sciences (IUNICS-IdISBa), University of the Balearic Islands, 07120 Palma de Mallorca, Spain; charles.santana@gmail.com

**Keywords:** chronic pain, machine learning, classification, questionnaires, QST

## Abstract

In the last decade, machine learning has been widely used in different fields, especially because of its capacity to work with complex data. With the support of machine learning techniques, different studies have been using data-driven approaches to better understand some syndromes like mild cognitive impairment, Alzheimer’s disease, schizophrenia, and chronic pain. Chronic pain is a complex disease that can recurrently be misdiagnosed due to its comorbidities with other syndromes with which it shares symptoms. Within that context, several studies have been suggesting different machine learning algorithms to classify or predict chronic pain conditions. Those algorithms were fed with a diversity of data types, from self-report data based on questionnaires to the most advanced brain imaging techniques. In this study, we assessed the sensitivity of different algorithms and datasets classifying chronic pain syndromes. Together with this assessment, we highlighted important methodological steps that should be taken into account when an experiment using machine learning is conducted. The best results were obtained by ensemble-based algorithms and the dataset containing the greatest diversity of information, resulting in area under the receiver operating curve (AUC) values of around 0.85. In addition, the performance of the algorithms is strongly related to the hyper-parameters. Thus, a good strategy for hyper-parameter optimization should be used to extract the most from the algorithm. These findings support the notion that machine learning can be a powerful tool to better understand chronic pain conditions.

## 1. Introduction

Pain is a subjective perceptual phenomenon, which is mainly determined by emotional, cognitive, and sociocultural factors (e.g., mood, learning, attention, and beliefs) [1,2]. More importantly, pain is an emergent property of brain activity, in which learning and memory processes, along with associated plastic brain changes, may play a very important role, particularly when pain persists over time [3]. The International Association for the Study of Pain (IASP) defines chronic pain as pain that lasts more than three or six months [4,5]. Other symptoms, such as sleep disturbance, mood changes, and fatigue, are also associated with chronic pain syndromes [6,7]. These physical, cognitive, and emotional alterations clearly affect patients’ daily routines, leading to impairments of quality of life and disability.

Hence, the assessment and treatment of pain require a multidimensional approach that takes into account neurophysiological, psychological, and cultural aspects related to pain perception. As a result, several techniques and tools have been specifically developed or incorporated for measuring all of these multidimensional aspects of pain. Thus, for instance, surveys and self-report questionnaires [8,9], Quantitative Sensory Tests (QSTs) [10,11,12,13], genetic factors [14,15,16], physical activity patterns [17,18,19,20], Electroencephalography (EEG) [21], neuroimaging [22,23,24], and, more recently, functional near-infrared spectroscopy (fNIRS) [25] have been incorporated into studies of the emotional and cognitive factors that modulate pain. Eventually, these approaches are used to classify or differentiate groups, comparing patients with one chronic pain syndrome against pain-free controls or other chronic pain syndromes. Often, one study can present results for more than one tool or method, testing different cut-offs or applying it in different body regions. Usually, the different chronic syndromes do not share the same diagnostic approaches and, in most cases, the diagnosis of chronic pain is solely based on individuals’ self-reports. All these studies have expanded our comprehension of the neurophysiological mechanisms (central sensitization, brain plasticity) and psychosocial factors involved in the origin and maintenance of chronic pain. Nevertheless, more than 75% of patients do not receive an accurate diagnosis [26,27]. In addition, primary care providers show inappropriate attitudes and beliefs about pain and its treatment even after participating in continuous education programs [28].

In this context of inaccurate diagnosis and treatment of chronic pain, clinicians’ decisions could benefit from objective methods and criteria for better understanding and treatment of patients with chronic pain. In this regard, the US Food and Drug Administration (FDA) and the American Pain Society (APS) have proposed a multidimensional framework and operational diagnostic criteria for the major chronic pain conditions. Those criteria are composed of the patient’s historical data, questionnaires (self-reported), and psychophysical tests that determine pain features and pain thresholds. Furthermore, the Analgesic, Anesthetic, and Addiction Clinical Trial Translations, Innovations, Opportunities, and Networks (ACTTION) Framework [29] has suggested taking into account at least the following five dimensions: (1) core diagnostic criteria; (2) common features; (3) common medical and psychiatric comorbidities; (4) neurobiological, psychosocial, and functional consequences; and, finally, (5) putative mechanisms, risk factors, and protective factors. Expanding this framework, other studies have proposed evidence-based diagnostic criteria for specific chronic pain conditions [30,31,32,33,34,35,36].

Chronic lower back pain (CLBP) is one of the most frequently investigated syndromes and a major cause of work disability. The majority of the studies about CLBP use methods related to mobility measures [19,37,38,39,40] or electromyography [41,42,43]. Using the ACTTION framework, Markman and colleagues listed four items as the core diagnostic criteria for CLBP: (i) pain restricted to the lower back or with a referral pattern limited to the proximal legs, (ii) pain in the lower back on most days for at least three months and at least half of the days in the past six months, (iii) absence of neurological symptoms and deficit or symptoms in the lower extremities, and (iv) absence of tumors, infections, spondylolisthesis Grade 2 or higher, acute vertebral fractures, or other identifiable primary causes of lower back pain. This core is complemented with the other dimensions, which include a set of comorbidities and other syndromes that may be associated with CLBP, including other common chronic pain syndromes, such as fibromyalgia (FM).

The American College of Rheumatology (ACR) recommends a combination of questionnaire data (the Symptom Severity Score) and the Widespread Pain Index (WPI) as classification criteria of fibromyalgia (FM) [6]. Over the years, the ACR’s criteria were updated and adapted for different languages [44,45], but in addition to their consolidation, new alternatives have been proposed [46]. Using the ACTTION framework, a Fibromyalgia Working Group defined three items as the core diagnostic criteria for FM: (i) multi-site pain, defined as six or more pain sites from a total of nine possible sites, (ii) moderate to severe sleep problems or fatigue, and (iii) multi-site pain plus fatigue or sleep problems must have been present for at least three months [32]. Complementary tenderness, discognition, and musculoskeletal stiffness are common features experienced by FM patients. In both FM and CLBP, patients may face pain sensitivity alterations, as well as mood disorders such as depression and anxiety.

Finally, it seems clear that complex and multidimensional classification problems could take advantage of machine learning techniques applied to clinical data for supporting clinical decisions [47,48,49,50,51]. In the context of pain and pain chronification, machine learning approaches have recently been applied to several pain syndromes [24,52,53,54], including fibromyalgia [55,56,57] and chronic lower back pain [58,59,60,61]. While traditional statistical analyses commonly make some a priori assumptions about the data model (e.g., normality) and about the relationships among variables (e.g., linearity), machine learning prioritizes a “distribution-free” context. Thus, machine learning algorithms can learn through the data, identifying more complex relationships among variables and selecting the model that best describes a problem [62]. The design of a machine learning application may include several steps, starting from the data selection and preprocessing until the correct evaluation and validation. The application’s performance is dependent on the correct implementation of these steps [63,64], especially the training process, where the model’s hyper-parameters are optimized [65,66]. In this current study, we assessed the sensitivity of different machine learning algorithms and datasets for classifying chronic pain syndromes along with investigating the effect of hyper-parameter optimization.

## 2. Materials and Method

### 2.1. Participants

The participants of the study were 338 pain-free controls (HC) (age: 40.66 +/− 15.46, females: 258) and 659 chronic pain patients (age: 50.55 +/− 10.50, females: 567). The chronic pain group (CP) was composed of 440 fibromyalgia patients and 219 chronic back pain patients. There was no significant difference in age between the groups. All chronic pain participants matched the IASP’s criteria [5] and/or the ACR’s criteria for fibromyalgia [6]. A relatively small number of patients reported the use of non-steroidal anti-inflammatory drugs (NSAIDs) or paracetamol ( *n* = 4), benzodiazepine (1–5 mg per day) (*n* = 3), and serotonin re-uptake inhibitors (*n* = 1). None of the selected participants used opiates, gabapentin, or pregabalin for pain treatment. The study was approved by the Research Ethics Committee of the Balearic Islands with the following codes: IB833/07PI and IB2268/14PI on 25 February 2015. The participants provided their written informed consent to participate in this study.

### 2.2. Data Acquisition

The Beck depression inventory II (BDI) [67], the State–Trait Anxiety Inventory (STAI) [68], and nine measures related to quantitative sensory tests (QSTs) of pain were analyzed in this study. The BDI and STAI are two questionnaires associated with depression and anxiety, respectively. These are disorders commonly correlated with chronic pain conditions [69,70,71,72,73]. The QST measures and body location included: pressure thresholds (index finger and wrist), heat threshold (index finger), and cold threshold (index finger, wrist, and elbow). In addition, the ratio between pain ratings and stimulus intensity applied was computed for pressure stimulus (index finger and wrist) and heat stimulus (index finger). All tests were applied on the dominant side. Heat and cold pain sensitivity were measured with a computer-controlled contact thermal stimulator (cold/warm plate AHP-301CPV, Teca, Schubert, IL, USA), while the pressure pain sensitivity was measured with a digital dynamometer using a flat rubber tip (1 cm^2^; Force One, Wagner Instruments, Greenwich, CT, USA).

### 2.3. Data Preprocessing

All data were previously normalized, and missing values were replaced by using multiple imputation by chained equation (MICE) [74]. Other imputation methods, such as the k-nearest neighborhood (k-NN) imputer, mean, and median, were also tested, but MICE was chosen because it preserved characteristics such as variance and correlation among variables. Subjects with more than one missing value were excluded, resulting in the number of participants cited previously. Finally, to avoid any kind of bias due to the position of the subjects inside the data, the subjects’ positions were shuffled.

### 2.4. Classifiers

Eight classifiers were compared to determine the one that presented the best results in a binary classification problem: chronic pain patients against controls. This set of classifiers includes simple linear classifiers as well as ensemble-based classifiers. Logistic regression (LR) and support vector classifiers (SVC) are two linear models. LR uses a logistic function to model the probabilities of an object being part of a specific class [75], while the SVC algorithm searches for a hyper-plane capable of classifying the objects by maximizing the distance between the hyper-plane and the data points. The k-NN classifier was also tested. The k-NN is a nonparametric technique where the class of an object is defined by the classes of k-other objects near it [76]. Another nonparametric method used was the dynamic tree classifier (DTC), which constructs a series of rules inferred from the data features [77]. The random forest classifier (RFC) and extra trees classifier (ETC) are ensembles of multiple DTCs in a unique classifier, where each tree predicts one class for the object, and the most predicted class is the final prediction made by the RFC [78] or ETC. The other classifiers used a multiple layer perceptron classifier (MPL), which is also known as neural network, and an extreme gradient-boosting classifier (XGBoost) [79]. Table 1 presents a list of all classifiers and the set of hyper-parameters.

In order to have a reference base for the chosen classifiers, dummy classifiers were used in this study. These classifiers do not perform any kind of learning. Their predictions are based on five strategies: a completely random guess following a uniform distribution (uniform); always predicting the most frequent class (most_frequent); always predicting the class that maximizes the class prior (prior); always predicting the HC class (constant = 0); always predicting the CP class (constant = 1); and predicting randomly but keeping the proportion of classes observed in the training data (stratified).

### 2.5. Training and Evaluation

In this study, we evaluated five datasets with the following compositions: (1) *age* dataset with only the information about participants’ ages; (2) *basic-wo-age* dataset with data from the BDI, STAI State (STAI-S), and STAI Trait (STAI-T); (3) *basic* dataset, adding the age information to *basic-wo-age*; (4) *qst* dataset with the nine QST measures; and, finally, (5) *all* dataset with all available data.

**Figure 1:**
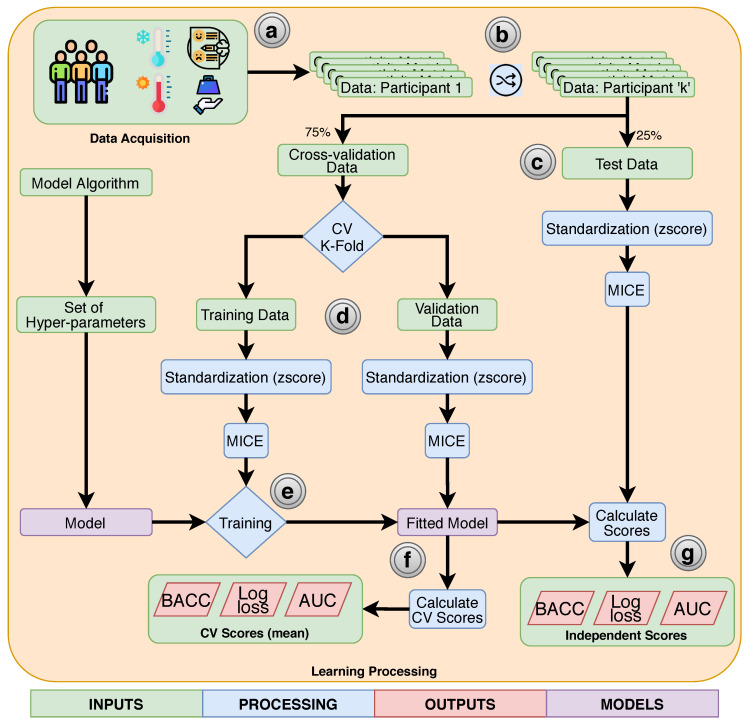
Flowchart describing the entire process of acquisition, preprocessing, processing, learning, and evaluation. (**a**) Participants answered the questionnaires and were evaluated using the sensorial tests of cold, heat, and pressure. (**b**) The learning process started shuffling the list of subjects’ data. (**c**) An independent test was selected to be used to compare the best-fitted models after hyper-parameter optimization; this set represents 25% of the total dataset and preserves the proportion of target classes. With the remaining 75%, we applied a five-fold cross-validation process to optimize the hyper-parameters (**d**). The cross-validation applied two equal pipelines for the training and validation data. These pipelines sequentially apply a standardization method, an imputation method (multiple imputation by chained equation (MICE)), and the model in a training context (**e**) or the fitted model in order to obtain the cross-validation scores (**f**). Finally, (**g**) the fitted model is evaluated using the independent test and the same pipeline used during the cross-validation.

For all classifiers, we applied a stratified k-fold cross-validation approach with k equals 5. In addition, we forced the cross-validation to select the same folds for all classifiers. All scores presented in this paper are related to the average of the cross-validation folds. Figure 1 describes the entire process of acquisition, preprocessing, processing, learning, validation, and evaluation. During the cross-validation process, we selected different combinations of hyper-parameters using a randomized search [80] until a maximum of one thousand combinations. This selection was replicated for each classification method and used during the training, validation, and evaluation processes.

We used three scores to evaluate and compare the experiments. First, we used the balanced accuracy (BACC), defined as the average of recall obtained on each class, which, in turn, is the proportion of actual positives that are predicted as positives. Imbalanced groups do not affect this accuracy score. Then, we applied the area under the receiver operating curve (AUC) of a classifier, which is equivalent to the probability that the classifier will rank a randomly chosen positive instance higher than a randomly chosen negative instance [81]. For the last, we used the cross-entropy loss or log-loss (LLOSS), which is defined as the negative log-likelihood of the true labels given the probabilistic classifier’s predictions. It can be interpreted as a measure of certainty, where a classifier that predicts a wrong class with a high probability is punished [82]. We calculate it using the probability of an instance belonging to a target class, and the values can range from 0 to +∞, where values close to 0 are better scores. Oppositely, for both the BACC and AUC, the values can range between 0 and 1, and values close to 1 indicate better classifiers. For all scores, we used CP as the predicted class.

## 3. Results

In total, this study trained and evaluated 26,130 models, including dummy classifiers. From this set of models, we excluded all models that displayed an LLOSS higher than 0.69. In a binary classification problem, this cut-off represents the value of LLOSS for a model that predicts all elements with probability equal to 0.5 (randomly). Values higher than this cut-off mean that the model is less reliable than a prediction by chance.

**Figure 2:**
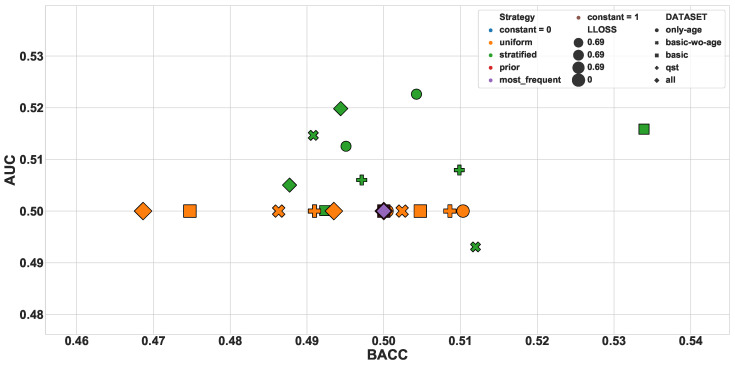
Baseline based on dummy classifiers.

**Figure 3:**
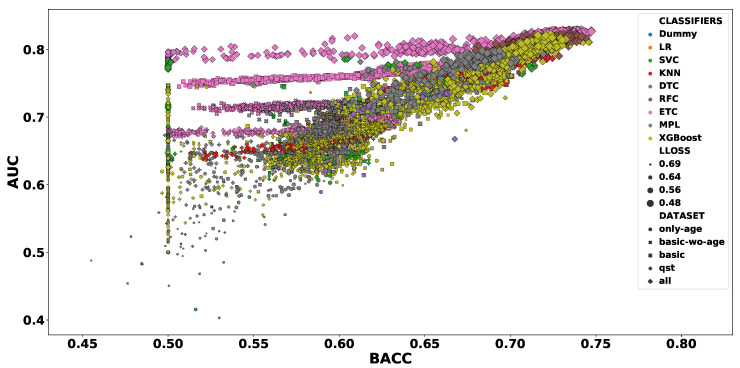
Dispersion (mean balanced accuracy (BACC) vs. mean area under the receiver operating curve (AUC)) of cross-validation scores. Each color represents one type of classifier, while the shape corresponds to the evaluated dataset. The size of each marker is inversely proportional to the value of log-loss (LLOSS; cross-validation mean); thus, bigger markers indicate more reliable classifiers.

**Figure 4:**
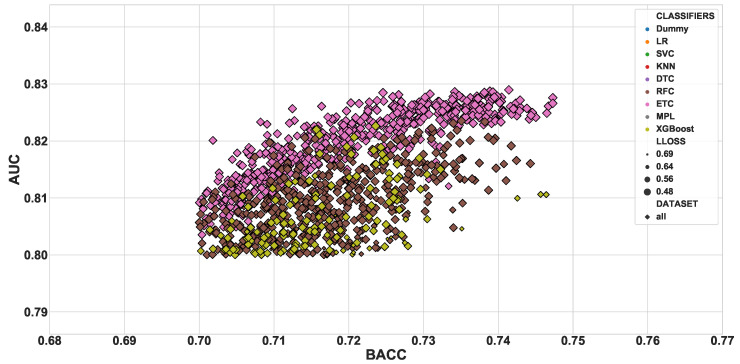
Close-up in the dispersion (mean BACC vs. mean AUC) of the cross-validation scores.

The dummy classifiers served as a baseline for the other classifiers. Figure 2 presents the values of BACC and AUC obtained for all dummy classifiers. As expected, the values orbited around the selection by pure chance (0.5). The other classifiers presented a wider range of values, varying from 0.455 to 0.747 for BACC and between 0.403 and 0.828 for AUC. Figure 3 shows the dispersion of results for all 26,130 models. In that figure, we can observe that the dataset *all* prevails among the high values. Focusing on the models of the top-right corner (BACC > 0.7 and AUC > 0.8), Figure 4 reveals that only this dataset is represented.

**Figure 5:**
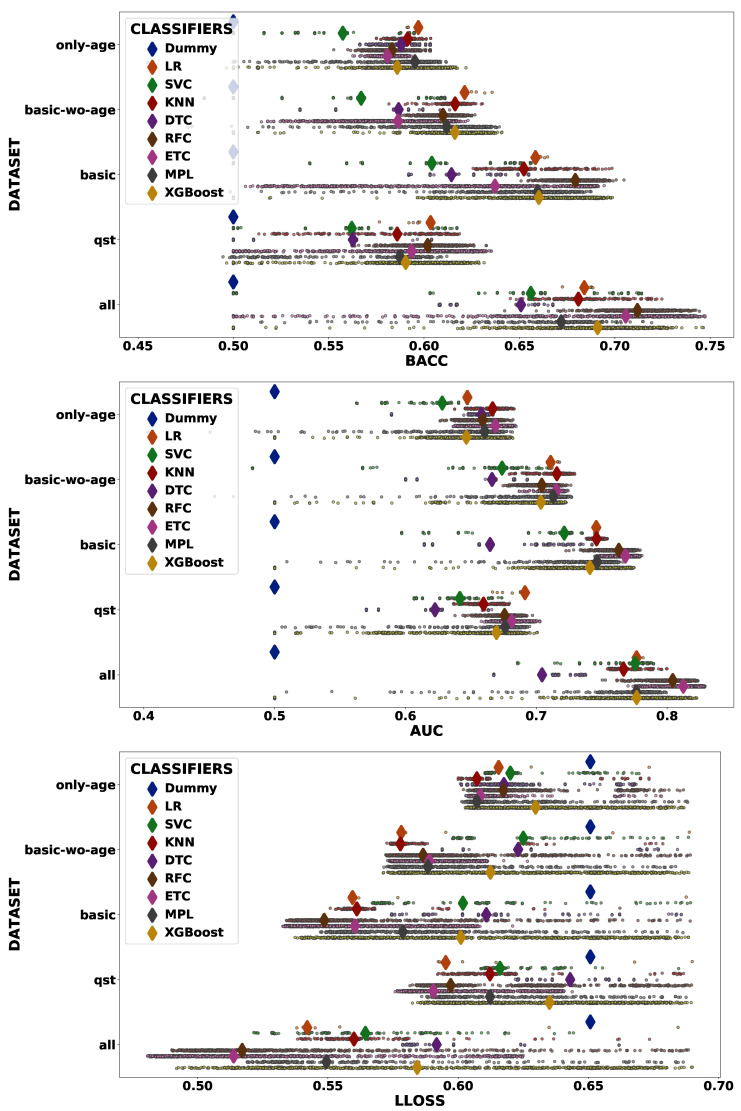
Strip plot for the BACC (**top**), AUC (**middle**), and LLOSS (**bottom**) grouped by dataset (vertical axis) and classifier type (colors). Each point represents one classifier with a specific hyper-parameter configuration, while the diamonds represent the medians of these points.

Figure 5 exhibits more details about the dataset selection in the classifier performance. In addition to the greater number of features, the *qst* had a worse performance compared to the *basic* dataset, with values closer to the *only-age* dataset. In addition, we can observe a very similar result when comparing the *basic* and *basic-wo-age* datasets, which indicates that age does not contribute with relevant information to the classification problem.On the contrary, when we evaluate the *all* dataset, the inclusion of *qst* features seems to import useful information, resulting in a notable increase in the classification performance of all classifiers.

Excluding the baseline, we can notice that while DTC had the worst performance, the classifiers based on an ensemble of classification trees presented the best performance overall. The superior performance of the ensemble-based classifier is reinforced when the number of features per dataset is increased. Another interesting point of this result is the spread of the ensemble-based classifiers, which indicates that these classifiers are more sensitive to the hyper-parameter selection. Due to the clear superiority of the *all* dataset, henceforth, we will only show results for that dataset.

### 3.1. Independent Test Scores

**Figure 6:**
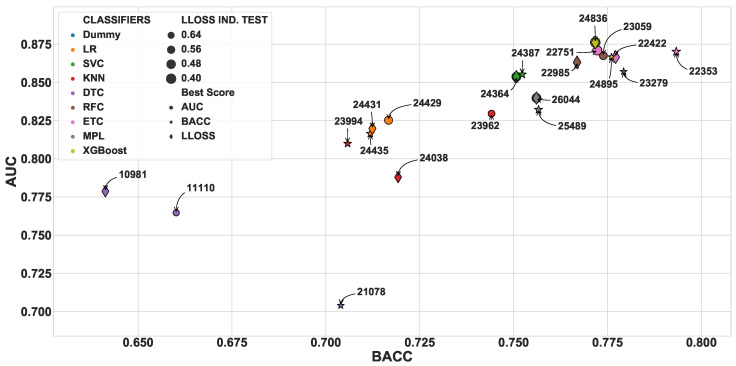
Independent test scores (BACC vs. AUC) for the best classifiers selected after the hyper-parameter optimization. The markers’ shapes represent the score used to select the best classifier, while the colors represent the classifier type. Similarly to the other figures, the marker size is inversely proportional to the LLOSS. The annotated numbers are the unique identifiers of each classifier. In this evaluation, only the *all* dataset was evaluated.

After the hyper-parameter optimization, we selected the best classifier of each type based on the three scores: AUC, BACC, and LLOSS. Then, this set of 24 well-tuned classifiers was evaluated using the independent test to determine which of them performed the classification task better. In Figure 6, we present the values of BACC versus AUC for each of the best classifiers. In addition, the marker size is inversely proportional to the value of LLOSS. This result confirms the pattern found during the hyper-parameter optimization phase: The ensemble-based classifiers have the best performance in the classification problem. The ETC classifier with id 22353 provided the highest values of BACC (0.793), while an XGBoost classifier (id: 24836) presented the best AUC (0.876), as well as the lowest value of LLOSS (0.423). In addition, the classifiers with the higher AUC values (>0.85) presented good values of precision (0.81) and recall (0.90). Finally, analyzing the given probabilities for the independent test, we can also observe that only a small number (2%) of subjects were wrongly classified with a probability higher than 0.8.

### 3.2. Model Interpretation

**Figure 7:**
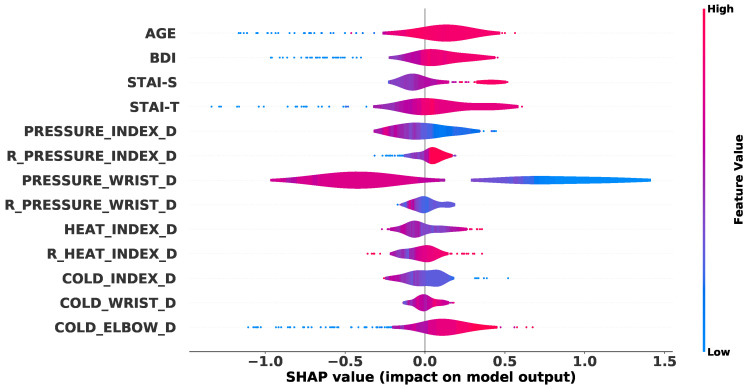
Summary explanation for the best extra trees classifier (ETC). Each dot represents one of the samples contained in the independent test. On the horizontal axis, the values indicate how much each feature (on the vertical axis) contributes for an HC or CP classification with negative and positive values, respectively. The color represents the feature value. The prefix “R_” among the quantitative sensory test (QST) variables indicates that they correspond to the ratio between pain ratings and stimulus intensity.

One of the main challenges in machine learning solutions is to understand how the algorithms make predictions. Predictions based on factors that are supported by a knowledge-based theory are more reliable and preferable in the context of problems involving humans. In that direction, we used shapely additive explanations (SHAP) to obtain an approximation of how our trained algorithms predict chronic pain conditions. In Figure 7, we display a summary of the best ETC algorithm using SHAP. The explanation shows that the algorithm associates higher values of age, BDI, STAI-S, STAI-T, and pain ratios (any location) with CP patients. In the same way, higher values of pressure thresholds are associated with HC participants. These findings provide more reliance on the appropriateness of the machine learning algorithm to separate participants into healthy subjects and patients with chronic pain.

**Figure 8:**
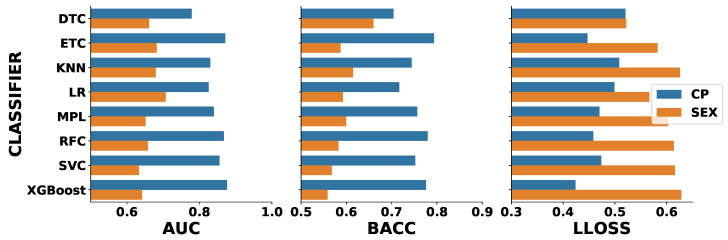
Independent test scores for the best classifiers compared against a hypothetical case where the classifiers were predicting the sex and not the chronic pain.

Finally, due to the fact that our target groups were not well balanced regarding gender (with a predominance of females in the CP group), we evaluated the best set of algorithms to determine if they were classifying participants’ sex instead of pain vs. healthy group. This could occur if the algorithms learned based on some sex information hidden in the features. To check this hypothesis, for the independent test, we calculated all the scores (AUC, BACC, LLOSS) using the sex information, encoded as 0 (male) and 1 (female) instead of the groups HC (0) and CP (1). In case our hypothesis is correct, we expect to have an equal or better performance of the algorithms using this new reference. In Figure 8, we show the results for both references.

We notice that AUC and BACC values are reduced when sex is used as a reference. This result becomes clearer when looking at the ensemble-based classifiers, the ones that had the best results overall. Another important point to note is the LLOSS score; we can see that the reliability of the classifiers degraded with an increase in the values of LLOSS for this new reference. All these factors gave us the confidence to exclude the cited hypothesis in all scenarios.

## 4. Discussion

In this paper, we assessed different machine learning algorithms to classify participants into chronic pain patients or healthy controls based on self-report and pain sensitivity data. In contrast to previous studies (e.g., Nijeweme-d’Hollosy and colleagues [83]), here, we compared different machine learning algorithms in a binary classification problem. From a methodological point of view, our study expands the study of Nijeweme-d’Hollosy by including more algorithms and a hyper-parameter optimization analysis, thus demonstrating that the success of classifiers depends on this optimization process.

The findings of this study provide evidence that the hyper-parameter optimization is an important part of the work when using machine learning algorithms. The results are very sensitive to the set of hyper-parameters chosen, which can result in a variation of more than 10% in BACC. Based on the studies presented at the Conference of Neural Information Processing Systems meeting in 2019 (NeurIPS2019) and the International Conference on Learning Representations (ICLR), grid-search and manual optimization are the most common approaches for selecting the best hyper-parameters [84]. While grid-search can consume too many resources, manual optimization can be limited by the researcher experience. In that context, randomized search has been proven to be a good choice, allowing a more broad search compared with manual optimization, but with a reduced cost compared with a grid-search [80].

In the present study, the *qst* dataset was the biggest dataset (isolated) with nine features. Nevertheless, this dataset had a poor performance if compared with the *basic* dataset. Age, BDI, STAT-T, and STAI-S were the four features comprising the *basic* dataset; this collection of features had three types of information (age, depression, and anxiety), while the *qst* features described the same kind of information: pain sensitivity. Thus, another important finding of this study was that a more divergent dataset could improve the chronic pain classification and that the number of features by itself could not correspond directly with performance. On the other hand, a dataset with a poorer performance would still improve a superior dataset, which was the case when the *basic* dataset was unified with *qst*.

Finally, only information about patients’ age did not provide better performance than the baseline, and did not change the performance of the *basic* dataset. However, age information had some impact on the model outputs when the *all* dataset was considered. Thus, similarly to the hyper-parameter optimization problem, the training features should be chosen carefully. This feature selection can be based on previous knowledge of the problem, usually provided by some experts or by other studies. In addition, a sensitive experiment or other feature selection technique should be applied to select or confirm the most informative set of features.

In the present study, the best algorithms presented a fair to good performance overall, with values of balanced accuracy and AUC of around 0.75 and 0.85, respectively. Furthermore, the values of precision and recall indicated that the model produces more false negatives than false positives. In the context of our classification problem, the false positives were more acceptable, since they would further pass by a more complex exam to confirm or reject the prediction.

In the previous literature, other studies for classifying into chronic pain patients and healthy controls have shown higher performance rates. For instance, Hidalgo and colleagues presented a study using eight cameras and kinematic analysis to differentiate CLBP patients. In that study, the best scenario reached an AUC of 0.95 [18,19]. With similar performance, Jones and colleagues used RNA analysis and identified 10 candidate genes that could be used to predict FM with an AUC equal to 0.931 [85]. These studies presented more sophisticated and complex methods that are not commonly available in primary care. On the other hand, they could be used as complementary exams. Other studies also used simpler methods like questionnaires [86] or cost-saving sensorial tests [11].

Using common pegs and comparing against standardized algometers, Camara and colleagues also found that the pegs can have a higher AUC (0.81) in classifying non-malignant musculoskeletal pain [11]. Based on a Multidimensional Health Assessment Questionnaire (MDHAQ), Gibson and colleagues developed a simple fibromyalgia assessment screening tool to support the identification of FM in patients with other rheumatic diseases [87]. Different subsets of the MDHAQ were analyzed, and the *symptom checklist* exhibited the best AUC (0.926). This checklist has historical information about the patient in the last month. Among the sixty items, some variables were correlated with FM symptoms, such as sleeping disorders and, more directly, pain at different body sites. Isolating the variables related to anxiety and depression, the values of AUC decreased to 0.716 and 0.745, respectively.

The feature set of our study did not include any information about non-induced pain. Our objective was to avoid any kind of data leakage. Variables about persistent pain, such as the pain felt in the last week or daily, can be directly correlated with the predicted variable (CP or HC). The algorithms could take advantage of that data leakage and focus only on the pain variable during the learning process. That could affect our evaluation of the learning capacity of the algorithms. In future analyses, we understand that some information about persistent pain should be included.

Excluding the data leakage hypothesis and based on the shapely additive explanations (SHAP), we can perceive how each feature contributes to the prediction. Besides the fact that the *only-age* dataset did not present good results, the SHAP analysis indicates that younger subjects have a higher chance of being classified in the healthy control group. Following the same analyses, the algorithm learned that subjects with depression (high BDI values) had a higher chance of belonging to the chronic pain group than participants without depression. This feature seems to be supported by previous literature on chronic pain, where several studies have linked chronic pain to depression [69,70,71,72,73]. A similar interpretation could be observed on anxiety (STAI variables), with high values of anxiety being related to chronic pain conditions [88,89]. Finally, data about pain sensitivity variables seem to indicate two relevant issues: (1) Subjects with higher pressure thresholds had a higher probability of being a healthy control; and (2) when a participant presented a high ratio between the subjective pain reported and the objective pressure applied during the QST, there was also a high probability of being classified as a chronic pain patient. These two facts are also supported by previous studies, indicating greater sensitivity to pain in chronic pain patients than in healthy controls [13,90,91,92]. The other QST features presented the same pattern [93,94,95], but with a smaller contribution to the prediction. Summarizing, the findings from the present study revealed that: (a) Ensemble-based classifiers outperformed the other classifiers, (b) the selection of the hyper-parameter values and features plays an important role in the learning process, and, finally, (c) the classifiers presented in this study were able to learn and make decisions based on information that is supported by the literature of chronic pain.

In a clinical context, the findings of the present study reinforce the reliability of decision-support systems based on machine learning approaches for chronic pain classification. This approach could provide faster, cheaper, and, consequently, more accessible diagnoses for chronic pain patients.

Nevertheless, this study has some limitations. The sample of patients was restricted to a small part of the Spanish population, making necessary the validation of the method for other populations where the psychological response to chronic pain may differ [96,97], affecting the score of the questionnaires used in this study. Moreover, as shown by Botvinik-Nezer and colleagues, different data analysis pipelines could generate different results using the same data [98]. Thus, more validations are necessary to validate the machine learning approach in a more extensive population and with different data analysis pipelines before a translation to clinical practice can be made.

## 5. Conclusions

Machine learning techniques have been presented as an efficient option to support the solution of complex problems. Ultimately, the correct application and success of such techniques rely on the quality of the data passed to the algorithm as well as some learning process parameters. In health sciences, these applications have helped researchers to understand and to identify complex syndromes such as neoplasms, Alzheimer’s disease, and schizophrenia. Influenced by biological, social, and psychological factors, chronic pain is a complex syndrome that could take advantage of machine learning algorithms. As shown in this study on chronic pain classification, the success of these machine learning algorithms is tightly correlated with the amount of processed information, the hyper-parameter optimization, and the class of algorithms used. In brief, well-tuned algorithms based on an ensemble of classifiers present a higher chance of success. Nevertheless, this study has some limitations: To expand and reinforce the results, data from different sources and other preprocessing techniques must be evaluated.

## Figures and Tables

**Table 1:** List of tested classifiers and their corresponding sets of hyper-parameters and values. For more information about each hyper-parameter, please visit https://scikit-learn.org/stable/modules/classes.html.

Classifier	Set of Hyper-Parameters	Number of Combinations Evaluated
Dummy Classifier	strategy = [’constant’, ’uniform’, ’stratified’, ’prior’, ’most_frequent’]; constant = [0, 1];	10
Logistic Regression	solver = [’liblinear’]; penalty = [’l1’, ’l2’]; C = [$1\times 10^{-4},\;1\times 10^{-3},\;1\times 10^{-2},\;1\times 10^{-1},\;5\times 10^{-1},\;1.0,\;5.0,\;1\times 10^{1},\;1.5\times 10^{1},\;2\times 10^{1},\;2.5\times 10^{1}$]; dual = [True, False];	22
SVC	kernel = [’rbf’, ’poly’]; tol = [$1\times 10^{-5},\;1\times 10^{-4},\;1\times 10^{-3},\;1\times 10^{-2},\;1\times 10^{-1}$]; C = [$1\times 10^{-3},\;1\times 10^{-2},\;1\times 10^{-1},\;5\times 10^{-1},\;1.0,\;5.0,\;1\times 10^{1},\;1.5\times 10^{1},\;2\times 10^{1},\;2.5\times 10^{1}$];	110
K Neighbors Classifier	n_neighbors = range(1, 101, 1); weights = [’uniform’, ’distance’]; p = [1, 2];	2000
Decision Tree Classifier	criterion = [’gini’, ’entropy’]; max_depth = range(1, 19, 1); min_samples_split = range(2, 21, 1); min_samples_leaf = range(1, 21, 1);	1000
Random Forest Classifier	n_estimators = [3, 6, 9, 12, 15, 18, 100]; criterion = [’gini’, ’entropy’]; max_features = range($5\times 10^{-2},\;1,\;5\times 10^{-2}$); min_samples_split = range(2, 21, 1); min_samples_leaf = range(1, 21, 1); bootstrap = [True, False];	1000
Extra Trees Classifier	n_estimators = range(100, 500, 50); criterion = [’gini’, ’entropy’]; max_features = range($5\times 10^{-2},\;1,\;5\times 10^{-2}$); min_samples_split = range(2, 21, 1); min_samples_leaf = range(1, 21, 1); bootstrap = [True, False];	1000
MPL	hidden_layer_sizes = range(5, 100, 5); solver = [’lbfgs’, ’adam’, ’sgd’]; learning_rate = [’adaptive’, ’invscaling’, ’constant’]; learning_rate_init = [$1,\;1\times 10^{-1},\;1\times 10^{-2},\;1\times 10^{-3}$]	684
XGB Classifier	n_estimators = range(100, 500, 50); max_depth = range(1, 11, 1); learning_rate = [$1\times 10^{-3},\;1\times 10^{-2},\;1\times 10^{-1},\;5\times 10^{-1},\;1.0$]; subsample = range($5\times 10^{-2},\;1,\;5\times 10^{-2}$); min_child_weight = range(1, 21, 1); nthread = [1];	1000
